# Supplementary material for: An intersectional gene regulatory strategy defines subclass diversity of C. elegans motor neurons
Source: eLife. 2017 Jul 5;6:e25751. doi: 10.7554/eLife.25751 (PMC5498135; doi:10.7554/eLife.25751)
Supplement: Figure 1—source data 1. — (extracted from www.wormwiring.org). <> = gap junction (electrical synapse), > = chemical synapse, bwm = body wall muscle, MSN = male-specific neuron (all such connections lettered in blue). Note that only some subclass-specific connections are non-dimorphic (green), while most are sexually dimorphic (red). Only connections represented by >3 serial sections are shown. The extent of dimorphism of the VB1 neurons is not currently known because of absence of available male data. DOI: http://dx.doi.org/10.7554/eLife.25751.003 [file elife-25751-fig1-data1.docx]

**Figure 1 Source data 1.** The source data for the connectivity data shown in Fig.1B is listed below. “**<>”** indicates electrical synapse. “**>**“ indicates the direction of a chemical synapse. **MSNs** = male specific neurons. **bwm** = body wall muscle.

|  |  | **9 DA (DA1 - DA9)** | **7 DB (DB1-DB7)** | **12 VA (VA1-VA12)** | **11 VB (VB1-VB11)** | **11 AS (AS1-AS11)** | **3 SAB** |
| --- | --- | --- | --- | --- | --- | --- | --- |
| **Electrical synapse (<>)** | Shared by all | DA<>AVA for all | DB<> AVB for all | VA<>AVA for all | VB<>AVB for all |  | 3SAB<>AVA for all |
|  | Subclass-specific | DA8<>DVB |  |  |  |  |  |
|  |  | DA9<>PVR |  |  |  |  |  |
|  |  | DA9<>PHC |  |  |  |  |  |
|  |  | DA9<>PDA |  |  |  |  |  |
| **Chemical synapse (>)** | Shared by all | DA>D-type for all | DB>D-type for all | VA>D-type for all | VB>D-type for all | AS>D-type for all | 3SAB>bwm |
|  |  | DA>bwm for all | DB>bwm for all | VA>bwm for all | VB>bwm for all | AS>bwm for all |  |
|  | Subclass-specific  MN subclass is **pre**-synaptic | DA9>PDA (male-specific) |  | VA11>MSNs (PVV, PVY) | VB1>SAA | AS11>PDB (male-specific) |  |
|  |  |  |  | VA12>MSNs (PVV, PVZ) | VB1>RIF |  |  |
|  |  |  |  | VA12>PVC (not sex- specific) | VB1>RIM |  |  |
|  |  |  |  | VA12>DA8/DA9 (not sex- specific) | VB10>MSN (PVY) |  |  |
|  |  |  |  |  | VB11>MSN (PVV) |  |  |
| **Chemical synapse (>)** | Shared by all | AVA>DA for all | DA>DB for all | AVA>VA for all | VA>VB for all | AVA>AS for all | AVD>3SAB |
|  |  |  | PVC>DB for all | VD>VA for all | VD>VB for all |  | AVE>3SAB |
|  |  |  |  |  |  |  | AVL>3SAB |
|  | Subclass-specific  MN subclass is **post**-synaptic | DVB>DA8 (herm-specific) | MSNs (PVY, PVX, RnB)>DB7 | PHB>VA12 (herm-specific) | RIM>VB1 | PDB>AS11 (male-specific) |  |
|  |  | MSNs>DA8 (male-specific) |  | PHC>VA12 (herm-specific) | MSNs (PVV, PVY)>VB11 | PDA>AS11 (male-specific) |  |
|  |  | PHA>DA8 (NOT sex-specific) |  | MSNs (PVV et al.)>VA12 |  | PHC>AS11 (male-specific) |  |
|  |  | VA12>DA8 (NOT sex-specific) |  |  |  | MSNs (ray sensory)>AS11 |  |
|  |  | PHC>DA9 (herm-specific) |  |  |  |  |  |
|  |  | AVG>DA9 (male-specific) |  |  |  |  |  |
|  |  | MSNs (RnBs)>DA9 |  |  |  |  |  |
|  |  | VA12>DA9 (NOT sex-specific) |  |  |  |  |  |
